# Association between severe acute respiratory syndrome coronavirus 2 antibody status and reinfection: A case-control study nested in a Colorado-based prospective cohort study

**DOI:** 10.1016/j.pmedr.2023.102530

**Published:** 2023-12-01

**Authors:** Ingrid A. Binswanger, Komal J. Narwaney, Jennifer C. Barrow, Kathleen B. Albers, Laura Bechtel, Claudia A. Steiner, Jo Ann Shoup, Jason M. Glanz

**Affiliations:** a Institute for Health Research, Kaiser Permanente Colorado, Aurora, USA; b Colorado Permanente Medical Group, Denver, USA; c Department of Medicine, University of Colorado School of Medicine, Aurora, USA; d Department of Health Systems Science, Bernard J. Tyson Kaiser Permanente School of Medicine, Pasadena, USA; e Siemens-Healthineers, USA; f Department of Epidemiology, Colorado School of Public Health, Aurora, USA

**Keywords:** COVID-19 serological testing, COVID-19 epidemiology, Case-control study, COVID-19 vaccine, Infectious disease, Antibody

## Abstract

The association between the presence of detectable antibodies to severe acute respiratory syndrome coronavirus 2 (SARS-CoV-2) and SARS-CoV-2 reinfection is not well established. The objective of this study was to determine the association between antibody seronegativity and reinfection. Methods: Participants in Colorado, USA, were recruited between June 15, 2020, and March 28, 2021, and encouraged to complete SARS-CoV-2 molecular ribonucleic acid (RNA) and serology testing for antibodies every 28 days for 10 months. Participants with reinfections (positive SARS-CoV-2 RNA test ≥ 90 days after the first positive RNA test) were matched to controls without reinfections by age, sex, date of the first positive RNA test, date of the last serology test, and serology test type. Using conditional logistic regression, case patients were compared to control patients on the last serologic test result, with adjustment for demographic and clinical confounders. Results: The cohort (n = 4,235) included 2,033 participants with ≥ 1 positive RNA test, of whom 120 had reinfection. Among the 80 case patients who could be matched, the last serologic test was negative in 12 of the cases (15.0 %) whereas the last serologic test was negative in 77 of 1,034 (7.5 %) controls. Seronegativity (adjusted OR [aOR] 2.24; 95 % CI 1.07, 4.68), Hispanic ethnicity (aOR 1.87; 95 % 1.10, 3.18), and larger household size (aOR 1.15; 95 % 1.01, 1.30 for each additional household member) were associated with reinfection. Conclusions: Seronegative status, Hispanic ethnicity, and increasing household size were associated with reinfection. Serologic testing could be considered to reduce vaccine hesitancy in higher risk populations.

## Highlights

• In this Colorado-based study, 2,033 participants were recruited for monthly monitoring of SARS-CoV-2 infection and serology status.
• One hundred and twenty participants who previously had SARS-CoV-2 had a laboratory confirmed re-infection.
• Fifteen percent of the cases with re-infections were seronegative whereas 7.5% of the controls were seronegative.
• The odds of seronegativity were 2.24 times greater in cases with re-infection than controls.
• Hispanic ethnicity and larger household size were also associated with re-infection.

## 1 Introduction

In April 2020, the first serological tests to detect severe acute respiratory syndrome coronavirus 2 (SARS-CoV-2) immunoglobulins were approved by the U.S. Food and Drug Administration under Emergency Use Authorization (EUA). Before the introduction of the first COVID-19 vaccines (Pfizer and BioNTech BNT162b2, Moderna mRNA-1273, and Janssen JNJ-78436735), public demand for testing to assess natural immunity to SARS-CoV-2 infection was robust. Since vaccines and boosters became available, it has been suggested that serological testing could be used to gauge individuals’ vaccine response and subsequent vaccine-induced immunity, or immunity at the population level. (World Health Organization, 2020, Bonanni et al., 2021, West et al., 2021) However, epidemiological data on the association between serologic results and SARS-CoV-2 infection and reinfection risk are lacking, and the Centers for Disease Control and Prevention (CDC) and the Infectious Diseases Society of America have cautioned against using the tests to influence vaccine decisions or other preventive behaviors. (Centers for Disease Control and Prevention, 2022a, INFECTIOUS DISEASES SOCIETY OF AMERICA. , 2020) This may be in part because prior serological research has been limited by selected samples, small sample sizes, the inability to capture asymptomatic reinfections, and short follow-up (Addetia et al., 2020, Edelstein et al., 2022, Fox et al., 2022, Hall et al., 2021, Lustig et al., 2021, Malhotra et al., 2022, Ren et al., 2022, Selhorst et al., 2021, Deeks et al., 2020, Jeffery-Smith et al., 2021). Additionally, predominant SARS-CoV-2 variants rapidly evolved (e.g., Alpha before May 2021; Delta May - December 2021; and Omicron after December 2021 in Colorado) (Colorado Department of Public Health and Environment, 2022), and few prior studies evaluated the association between antibody status and reinfection during the Omicron (B.1.1.529, BA.1) phase of the pandemic when reinfections were more commonly reported than during the Alpha (B.1.1.7) and Delta (B.1.617.2) phases (Bean et al., 2022).

To determine the potential utility of commercially available serologic testing for SARS-CoV-2 in preventive interventions, we conducted a case-control study nested in a population-based, longitudinal cohort of patients to examine the association between serologic status and the incidence of SARS-CoV-2 reinfection. We hypothesized that seronegative status would be associated with an increased risk of reinfection.

## 2 Methods

### 2.1 Study Design, Setting, and data sources

**Fig. 1:**
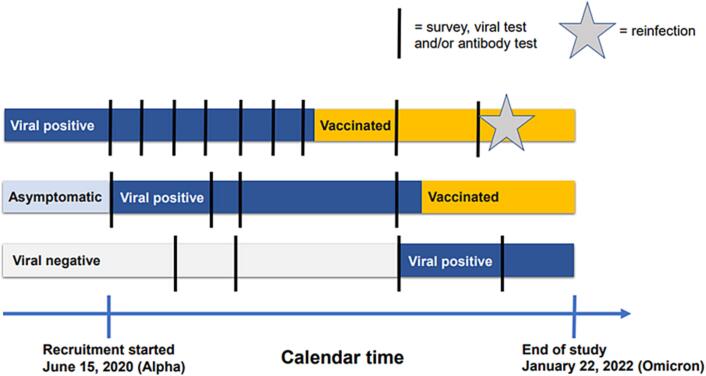
Illustrative depiction of the design of the longitudinal, population-based cohort.

**Fig. 2:**
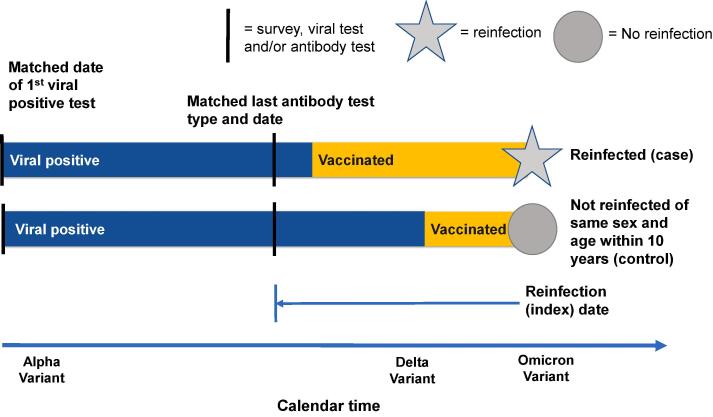
Illustrative depiction of the case-control design used for the study analysis.

We conducted a case-control study nested in a cohort of study participants recruited from Kaiser Permanente Colorado (KPCO), a healthcare delivery system integrated with an insurance plan comprised of employer-based, individual, Medicare, and Medicaid plans. A nested case-control design allowed us to match on time and serologic test type (Fig. 1 and Fig. 2), which was necessary given the rapid evolution of variants and tests during the unique study period. Data were derived from serial patient surveys, prospectively collected serologic and ribonucleic acid (RNA) samples, and electronic health records (EHR), including a COVID-19 patient registry. Data derived from the EHR included demographics, health plan enrollment dates, insurance type, laboratory results, immunization records (including data derived from state immunization registries), medication dispensations, diagnoses using International Classification of Diseases-10 codes, tobacco use, presumptive and confirmed SARS-CoV-2 infections, hospital utilization, and vital status.

### 2.2 Participant recruitment into the cohort and procedures

We recruited participants into a prospective survey and testing cohort who had suspected or confirmed SARS-CoV-2 infection between March 1, 2020, and March 28, 2021. Potential participants had a positive SARS-CoV-2 RNA or serologic tests in the EHR, or presumptive COVID-19 cases documented in the COVID-19 patient registry, regardless of whether they were ambulatory or hospitalized. Hispanic and Black/African American patients were oversampled to account for low response rates. We also recruited randomly sampled patients of similar age, sex, and race/ethnicity with no evidence of previous SARS-CoV-2 infection. Eligibility criteria included being a KPCO member, age ≥ 18, and having an available email or mobile number. Exclusion criteria included opting out of research, participating in the pilot survey, and needing a non-English language interpreter.

Eligible cohort members were recruited from June 15, 2020, to March 28, 2021, using text messages and emails linking to online consent documents. After consenting, participants completed baseline surveys and up to 10 follow-up surveys every 4 weeks, with follow-up procedures ending on January 22, 2022. Surveys were administered online using Research Electronic Data Capture (REDCap) (Harris et al., 2019) and included existing items from prior surveys and new items developed by the research team, reviewed by members of the KPCO Emergency Operations Command Center, and pilot tested. Baseline surveys included items about race, ethnicity, household size (von dem Knesebeck et al., 2018, Nguyen et al., 2011, Abrams and Szefler, 2020, Huijts et al., 2017), smoking, and vaping (Centers for Disease Control and Prevention, 2019), occupational categories, and behaviors and beliefs assessed using 5-point Likert scales (1 = strongly disagree; 5 = strongly agree). Follow-up surveys inquired about vaccine receipt and whether participants were told by a clinician that they had experienced coronavirus disease 2019 (COVID-19) or SARS-CoV-2 infection, regardless of whether they were tested for SARS-CoV-2.

At the end of each survey, participants were instructed to obtain SARS-CoV-2 molecular (viral RNA) testing and serologic (antibody) testing and, between surveys, RNA testing if they developed COVID-19 symptoms. Participants could obtain testing even if they did not complete surveys. Symptomatic participants, and participants with positive RNA tests within 10 days, were asked to skip serologic testing that month to protect laboratory staff from unnecessary exposure. Scheduling, clinical management, testing procedures (collection of nasopharyngeal, anterior nares, or oropharyngeal specimens for SARS-CoV-2 molecular testing and phlebotomy for SARS-CoV-2 serology), and laboratory assays (SARS-CoV-2 molecular analysis and serology) were conducted by KPCO clinical operations and laboratory services. Four serologic tests were in clinical use at KPCO during the study period: Abbott Architect SARS-CoV-2 nucleocapsid IgG test (COV-2 test), Siemens ADVIA Centaur SARS-CoV-2 (COV2T) Spike Total IgG/IgM, Siemens ADVIA Centaur Spike IgG COV2G, and Siemens ADVIA Centaur sCOVG Spike IgG (Appendix Table A1) (Bryan et al., 2020). SARS-CoV-2 molecular testing results were mostly from the transcription-mediated amplification (TMA) assay for the qualitative detection of SARS-CoV-2 by Aptima on the Panther (Hologic, Inc., San Diego, CA), which does not distinguish between viral variants nor quantify viral load. Participants were encouraged to receive COVID-19 immunizations as they became available. There was no compensation for participation other than receiving testing results.

The KPCO Institutional Review Board approved the study, and the study met the institution’s guidelines for protection of human subjects concerning safety and privacy. This study was registered in ClinicalTrials.gov (NCT05365750).

### 2.3 Case patients

Within the cohort, case patients had a reinfection, defined as a positive SARS-CoV-2 RNA test ≥ 90 days after the first positive RNA test, based on CDC guidance (Yahav et al., 2021). Case patients had to have serology completed between the infections. The date of the RNA test indicating reinfection was the index date. The serology test result closest in time before the reinfection was used to define the exposure.

### 2.4 Control patients

Control patients had a primary infection and serologic tests that were conducted between the primary infection and the index date but no evidence of reinfection by the index date. A control patient could be matched to more than one case if they had more than one eligible serologic test. Case patients were 1:20 matched to controls (representing unique participant-serologic test date combinations) by factors that could affect the serologic result and/or risk of reinfection: age (±10 years), sex, serologic test date (±30 days), serologic test type, and positive RNA test date (±30 days). (Bailie et al., 2022) A SAS® macro using the greedy matching algorithm (https://bioinformaticstools.mayo.edu/research/gmatch/) randomly sorted and sequentially matched case patients to the closest control (nearest neighbor) to prevent bias. The primary exposure was a seronegative test result after primary infection and closest in time to the index date.

### 2.5 Covariates

The following covariates were considered: insurance type, race, ethnicity, tobacco use or vaping (Qureshi et al., 2022), healthcare or frontline employment, household size, diagnoses associated with immunosuppression or chemotherapy (Centers for Disease Control and Prevention, 2022b), modified Charlson comorbidity index score (Deyo et al., 1992, Quan et al., 2005), and receipt of ≥ 1 dose of a COVID-19 immunization (survey-reported or documented) before the index date. Since protective behaviors and beliefs could be associated with reinfection risk and the propensity to adhere to study testing, we considered a composite score of the responses to 10 baseline behaviors and beliefs as a covariate.

### 2.6 Case-Control analysis

Conditional multivariable logistic regression evaluated the association between seronegative serologic status and reinfection, controlling for all covariates, except for vaccination, which could have been part of the causal pathway. We evaluated clinically plausible interactions between seronegative serologic status and ethnicity, and between seronegative serologic status and immunocompromised status.

### 2.7 Sensitivity analyses

During the study period, participants may have been immunized against SARS-CoV-2 before the primary infection, before or after the serologic test, and between the primary infection and the reinfection/index date or after the reinfection/index date. Serologic results for the 3 anti-spike protein tests could have been influenced by immunization or infection or both, while serologic results for the anti-nucleocapsid test would not have been influenced by immunization. Furthermore, a prolonged time between serologic testing and the index date could have attenuated any associations between serologic status and reinfection. People who might otherwise have had a negative spike protein serology could have seroconverted after immunization (but before the index date) and would therefore have been “misclassified” as seronegative in the primary analysis. Due to these issues, we conducted sensitivity analyses: (1) excluding anti-nucleocapsid serologic results; (2) excluding participants who received their first immunization after an anti-spike protein test; (3) excluding participants who had their last serology collected ≥ 164 days before the index date (the median time between serology and the index date); and (4) by the timing of the serologic test relative to vaccination (not vaccinated, serologic test on the same date/after the first vaccine, and serologic test before the first vaccine) using an interaction term.

## 3 Results

### 3.1 Cohort description

**Fig. 3:**
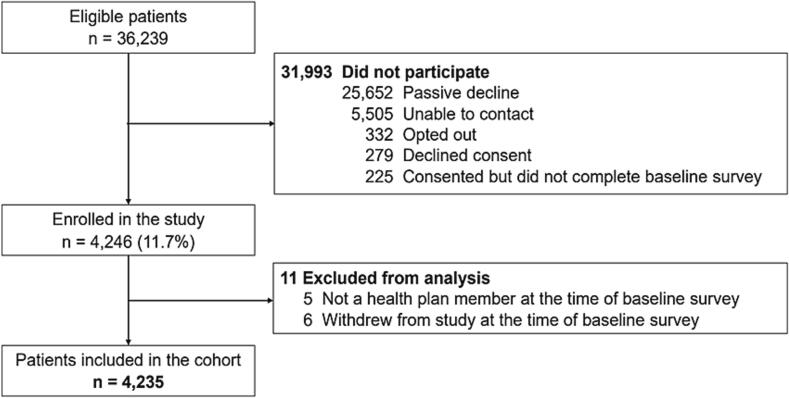
Study flow diagram.

Of 36,239 eligible members, 4,235 (11.7 %) consented to participate in surveys and testing procedures and were eligible for inclusion in the analysis (Fig. 3). Compared with non-respondents, EHR data indicated that survey and testing participants were older (mean 49.4 vs. 45.1 years) and more likely to be female (64.0 % vs. 54.2 %), non-Hispanic (76.6 % vs. 58.4 %) and white (71.0 % vs. 46.6 %), have Medicare (18.7 % vs. 14.8 %), and have a positive RNA test (46.7 % vs. 35.5 %) (Appendix Table A2).

In the survey, 20.0 % of participants identified themselves as Hispanic, 81.1 % identified as white, 14.4 % indicated they were healthcare or other front-line workers, and 9.5 % used tobacco or vaped nicotine (Table 1). The mean household size was 3.1 (SD = 1.6). One-quarter of survey participants were told by a clinician that they likely have or have had COVID-19 and 5.2 % (n = 219) were immunosuppressed. Mean baseline scores for protective behaviors and beliefs were 37.0 (SD 7.1) on scale of 10 to 50 (see Appendix Table A3 for individual item responses).

**Table 1:** Baseline self-reported characteristics of enrolled survey and testing cohort participants recruited between 2020 and 2021 and followed through 2022: Colorado, USA.

Characteristics	Participants (n = 4235)
Ethnicity, n (%)^a^	
Hispanic	848 (20.0)
Non-Hispanic	3375 (79.7)
Unknown	12 (0.3)
Race, n (%)^a^	
Black/African American	128 (3.0)
Asian/Pacific Islander	124 (2.9)
Native American	49 (1.2)
White	3435 (81.1)
Multiple	215 (5.1)
Other	217 (5.1)
Unknown	67 (1.6)
Household size including participant, mean (SD)	3.1 (1.6)
Tobacco use and/or vaping nicotine, n (%)^b^	402 (9.5)
Employment in health care and/or frontline occupation, n (%)	610 (14.4)
Told they were likely to have or have had COVID-19, n (%)	1054 (25.1)
Protective behavior and belief score, mean (SD); median (IQR)^c^	37.0 (7.1); 38.0 (33.0, 42.0)

Abbreviation: IQR, interquartile range; SD, standard deviation

a Self-reported survey information supplemented with information from medical record in case of missing responses.

b Assessed using survey questions on cigarette smoking and vaping. Supplemented with diagnosis codes and information from the social history in the medical record in the year prior to study enrollment.

c A higher score indicates more protective behaviors and beliefs.

At baseline, 1,976 (46.7 %) of 4,235 participants already had a positive RNA test indicating a primary infection, 373 (8.8 %) had a positive serologic test, 174 (4.1 %) had resolved COVID-19 illness, and 1,147 (27.1 %) were isolated or had clinical suspicion of infection without testing (Appendix Table A2).

Over the follow-up, participants had a mean [SD] of 4.1 [4.4] RNA tests and 4.2 [4.0] serologic tests. Follow-up testing identified 214 additional infections, for a total of 2,190 participants with laboratory-confirmed primary infection (Appendix Table A4). The estimated primary infection rate, calculated among the 565 participants who had no clinical or laboratory confirmed infections at baseline, was 0.64 per 100 person-months. Less than 5 of the 2,190 participants with a primary infection died due to COVID-19.

Among participants who had a primary infection, seropositivity ranged from 72.5 % to 93.2 % before vaccination and sero-reversion ranged from 1.1 % to 41.4 %, depending on the test type and its timing (Appendix Table A5). Seronegativity was more prevalent amongst immunosuppressed patients than those who were not (31.5 % vs 23.5 %).

Reinfections were identified in participants who had laboratory confirmed primary infection and ≥ 90 days of follow-up after the primary infection in which reinfection could have occurred. Among these 2,033 participants, nearly 6 % (n = 120) were reinfected for an estimated reinfection rate of 0.52 per 100 person-months. Most reinfections (103 of 120, 86.8 %) occurred during the Omicron phase and 74.1 % (n = 89) occurred among fully vaccinated individuals. No hospitalizations or deaths were associated with reinfection.

### 3.2 Case-control results

Of 120 cohort participants with reinfections, 92 had a previous serologic test, of whom 12 did not match any control on all matching variables. Thus, 80 case patients and 1,034 controls were included in the case-control analysis, and they differed by ethnicity, household size, and the last serologic status before the index date (Table 2). The last serologic test was negative in 15.0 % and 7.5 % of case patients and controls, respectively. Receipt of at least one vaccine dose was less common among case participants (78.8 %) than controls (85.8 %). Nearly 9 % of case participants but just 2.8 % of controls had a negative serologic test and had not been vaccinated.

**Table 2:** Demographic and clinical characteristics of case patients compared with matched controls: 2020–2022, Colorado, USA.

	Case patients (n = 80)	Controls (n = 1034)	p-value
Age, mean (SD), years^a^^,^^b^	52.7 (12.7)	54.4 (11.9)	0.54^b^
Female, n (%)^b^	60 (75.0)	761 (73.6)	1.00^b^
Ethnicity, n (%)^c^			0.009
Hispanic	32 (40.0)	252 (24.4)	
Non-Hispanic	48 (60.0)	782 (75.6)	
Insurance, n (%)^d^			0.75
Commercial	61 (76.3)	724 (70.0)	
Medicare	12 (15.0)	189 (18.3)	
Medicaid	4 (5.0)	46 (4.5)	
Private pay, self-funded, and others	3 (3.8)	75 (7.3)	
Household size, mean (SD)^e^^,^^f^	3.6 (2.3)	3.1 (1.6)	0.04
Tobacco use and/or vaping nicotine n (%)^g^, ^h^	4 (5.0)	47 (4.6)	0.87
Employment in health care and/or frontline occupation, n (%)^e^	18 (22.5)	153 (14.8)	0.38
Immunosuppressed n (%)	9 (11.3)	93 (9.0)	0.57
Modified Charlson Comorbidity Index, mean (SD)^i^	0.7 (1.1)	0.7 (1.1)	0.80
Received ≥ 1 vaccination dose prior to index date, n (%)	63 (78.8)	887 (85.8)	0.09
Time since the last serologic test, mean days (SD)	189.2 (136.2)	202.1 (126.7)	0.16
Last serologic test negative prior to index date, n (%)	12 (15.0)	77 (7.5)	0.01
Protective behavior and beliefs composite score, mean (SD)^e^^,^^j^	36.9 (35.1)	37.7 (37.2)	0.41
Antibody assay indexes among individuals with a positive serologic test, mean (SD)			
COV-2 nucleocapsid test	3.7 (1.7)	6.7 (2.7)	0.21
COV2T spike test (IgG/IgM)	9.5 (1.8)	8.0 (3.0)	0.14
COV2G spike test (IgG)	15.6 (7.1)	16.2 (6.7)	0.61
sCOVG spike test (IgG)	55.2 (38.4)	63.7 (41.9)	0.72

Abbreviations: COV-2, Abbott Architect SARS-CoV-2 nucleocapsid IgG test; COV2T, Siemens ADVIA Centaur SARS-CoV-2 Spike Total IgM/IgG test; COV2G, Siemens ADVIA Centaur SARS-CoV-2 Spike IgG test; sCOVG, Siemens ADVIA Centaur SARS-CoV-2 Spike IgG test, with wider range than COV2G; SD, standard deviation.

a Assessed at the index date.

b Case patients and controls were matched on age (±10 years), sex, first PCR positive date (±30 days), date the serologic test was conducted (±30 days) and the serologic test type; 12 case patients could not be matched to any control patient, for the remaining 80 cases, number of controls ranged from 1 to 20 (median = 14.0). Participants could serve as a control for more than one case if they had more than one serologic test; thus 532 of the controls were unique participants.

c Self-reported survey information supplemented with information from medical record in case of missing responses.

d If there was more than one insurance, the following hierarchy was employed: Medicaid, Medicare, commercial, and others.

e Assessed using survey data.

f Information was not missing for any cases but was missing for 10 controls. These were excluded from two analyses: univariate association of household size with reinfection and in the multivariable model.

g Assessed using survey questions on cigarette smoking and vaping. Supplemented with diagnosis codes and information from the social history in the medical record in the year prior to the exposure date.

h Assessed using diagnosis codes in the year prior and receipt of chemotherapeutic medications in the 6 months prior to the exposure date.

i Assessed in the year prior to study enrollment.

j Information was missing for 1 case and 51 controls and these were excluded from the univariate association of protective behavior score with reinfection analysis and in the multivariable model.

Vaccine status was not significantly associated with reinfection (OR 0.59; 95 % CI 0.32, 1.08). In adjusted analyses, the odds of reinfection among seronegative participants was 2.24 (95 % CI 1.07, 4.68) times that of seropositive participants. Hispanic ethnicity (adjusted OR [aOR] 1.87; 95 % 1.10, 3.18) and higher household size (aOR 1.15; 95 % 1.01, 1.30) were also significantly associated with reinfection (Table 3). Interactions between seronegative serologic status and ethnicity (p = 0.80), and seronegative serologic status and immunocompromised status (p = 0.83) were not statistically significant.

**Table 3:** Unadjusted and adjusted associations between SARS-CoV-2 seronegativity and reinfection (80 Case Patients and 1034 Controls), Colorado, USA 2020–2022.

	Univariate matched odds ratio (95 % CI)	Adjusted matched odds ratio (95 % CI)
Last serologic test negative prior to index date vs. positive	2.42 (1.21, 4.85)	2.24 (1.07, 4.68)
Ethnicity		
Hispanic vs. non-Hispanic	1.94 (1.18, 3.20)	1.87 (1.10, 3.18)
Insurance		
Medicaid vs. commercial	1.07 (0.36, 3.18)	0.83 (0.27, 2.58)
Medicare vs. commercial	0.75 (0.30, 1.86)	0.84 (0.31, 2.24)
High deductible vs. commercial	0.49 (0.06, 3.70)	0.68 (0.09, 5.34)
Private pay, self-funded, and others vs. commercial	0.56 (0.17, 1.84)	0.48 (0.14, 1.64)
Household size (each additional member)	1.14 (1.01, 1.28)	1.15 (1.01, 1.30)
Tobacco use and/or vaping nicotine vs. not	1.10 (0.37, 3.22)	1.17 (0.39, 3.56)
Health care/front-line worker vs. not	1.30 (0.73, 2.32)	1.21 (0.66, 2.21)
Immunosuppressed vs. not	1.25 (0.58, 2.67)	1.14 (0.48, 2.71)
Modified Charlson Comorbidity Index (each additional unit)	1.03 (0.83, 1.28)	1.04 (0.82, 1.33)
Received ≥ 1 vaccine dose prior to index date vs. no vaccination	0.59 (0.32, 1.08)	*
Protective behavior score (each additional unit)	0.99 (0.96, 1.02)	0.81 (0.49, 1.34)

Abbreviations: CI, confidence interval; N/A, not applicable.

* Not included in the model. Assessed as an interaction with the exposure in a sensitivity analysis.

### 3.3 Results of sensitivity analyses

Compared with the primary analysis, the association between seronegativity and reinfection was stronger when we (1) excluded participants with a nucleocapsid test (aOR 2.92; 95 % 1.44, 5.94), (2) excluded participants in whom vaccination occurred after the serologic test (aOR 4.23; 95 % CI 1.86, 9.62), and (3) excluded participants in whom the serologic test was done ≥ 164 days before the index date (aOR 3.96; 95 % CI 1.38, 11.37) (Appendix Table A6). In the interaction analysis (4), the association between seronegativity and reinfection was strong among those who were not vaccinated (aOR 4.19; 95 % CI 1.28, 13.76) and who had a vaccination before/on the same day as the serologic test (aOR 6.30; 95 % CI 1.66, 23.94). In contrast, no association between seronegativity and reinfection was observed among those who were vaccinated after the serologic test (aOR 1.08; 95 % CI 0.21, 5.48).

## 4 Discussion

In this nested case-control study, SARS-CoV-2 seronegative antibody status was significantly associated with reinfection. Sensitivity analyses support the robustness of findings, with stronger associations among patients with recent serology, vaccinated before serology, and without an intervening vaccination. These findings suggest serological testing may be useful to assess the risk of reinfection at the individual and population levels. The results also demonstrate modest independent associations of Hispanic ethnicity and household size with reinfection.

To date, clinical guidelines have suggested serology can be used in surveillance and the diagnosis of acute and post-acute COVID-19, but do not recommend using test results to elicit behavior change (Centers for Disease Control and Prevention, 2022a). However, given the significant acute and post-acute morbidity and mortality associated with reinfection (Al-Aly et al., 2022), these results support the use of serology to bolster preventive efforts. Hybrid immunity (previous infection plus vaccination) has been associated with reduced COVID-19 hospitalization rates compared with natural immunity alone (Nordstrom et al., 2022), yet high vaccine hesitancy has been documented in studies of COVID-19 recovered populations (Gerussi et al., 2021), perhaps due to low perceived risk of reinfection and its consequences. Serology could therefore be used to encourage immunization among COVID-19-recovered individuals who are seronegative but decline vaccines or boosters, particularly those at higher risk. Since Hispanic ethnicity and larger household size have also been associated with relatively high vaccine hesitancy and low vaccine acceptance rates (Nguyen et al., 2022), future research could evaluate whether communicating serology results influences vaccination behaviors among such previously infected, high-risk populations.

Our findings on the association between Hispanic ethnicity and reinfection is consistent with prior research showing that people of Hispanic ethnicity experience high rates of SARS-CoV-2 infection and mortality associated with COVID-19 disease (McCloskey et al., 2022, Mackey et al., 2021, Irizar et al., 2023). Studies suggest disproportionate infection rates may be related to disparities in access to care, cultural, political, and behavioral factors.

In this study, seronegativity may have been related to underlying immunocompromise, the timing of the test with respect to the primary infection, or poor test sensitivity (Al-Aly et al., 2022). Patients and clinicians may be concerned that convalescent seronegative status may suggest subclinical or unrecognized immunocompromise. Further research could evaluate whether further evaluation for immune deficiencies is needed in such patients.

By examining reinfections and including the Omicron phase, these findings extend prior research showing a negative correlation between IgG response and COVID-19 during prior phases (Molodtsov et al., 2022, Harvey et al., 2021, Alejo et al., 2022), the protective effects of infection on reinfection (Kojima et al., 2021), and high reinfection rates among people of Hispanic ethnicity (Slezak et al., 2021). In contrast to this study, a Boston study showed no association between antibody status within weeks of the primary infection and subsequent reinfection (Bean et al., 2022). The sensitivity of IgG serologic tests tends to be higher in the convalescent phase (21–100 days after symptoms start or positive RNA test) than the acute phase (Fox et al., 2022), and our case-control analysis included only seven observations with serologic testing done during the acute phase. In contrast to other studies (Slezak et al., 2021), we observed no significant associations between healthcare/frontline employment or immunocompromised status and reinfection, nor between baseline protective behaviors and beliefs and reinfection.

This study’s cohort had a higher reinfection rate than previous studies (Hall et al., 2021), approaching the primary infection rate we observed. The higher rate may be because we captured asymptomatic and mild reinfections, and followed the cohort into the Omicron phase, a variant associated with high transmissibility but milder infections. We also observed no hospitalizations or deaths associated with reinfection. The low morbidity and mortality may be because the cohort was community-based, with a large proportion remaining ambulatory with the primary infection, becoming immunized, and having access to COVID-19 treatment.

These results should be interpreted in the context of the study’s limitations and strengths. Participants differed from non-respondents, and we did not recruit Spanish speaking participants; therefore, the study sample characteristics should not be considered representative of the general population. Adherence to the study’s testing guidance was inconsistent. We encouraged regular on-site RNA testing but may not have captured some home-based antigen testing results towards the end of the study among participants who did not seek advice or care for COVID-19. Variable sensitivity and specificity of different serologic tests could have influenced our results. However, KPCO employed tests with EUA’s and were based on chemiluminescent immunoassays, which have better sensitivity and specificity than enzyme-linked immunoassays and lateral flow assays (Fox et al., 2022). Variable sero-reversion rates across tests could have been due to variable lengths of time between the primary infection and serology testing. The definition of a reinfection used could have identified viral persistence (recurrence), but the mean length of time between the primary infection and presumptive reinfection was 432 days (range 127–669) and there is little evidence of prolonged viral shedding (Cevik et al., 2021). We could not evaluate viral neutralizing activity and T-cell mediated immunity, although prior studies suggest these are correlated with IgG titers (Molodtsov et al., 2022). Nonetheless, this case-control study was conducted within a large and prospective cohort spanning three predominant COVID-19 variants, with long follow-up, and during a unique period during which near universal infection or vaccination had not yet occurred. We also encouraged testing among asymptomatic participants, which is a key strength since about 50 % of reinfections are asymptomatic (Sheehan et al., 2021).

## 5 Conclusion

This study demonstrated a strong association between serologic results and reinfection. Future research could explore whether serologic results could be leveraged to promote vaccination among people with primary infection who are vaccine hesitant and high-risk.

## Declaration of competing interest

The authors declare the following financial interests/personal relationships which may be considered as potential competing interests: Dr. Bechtel was employed at Kaiser Permanente Colorado at the time this study was conducted through November 2022 and is currently employed by Siemens Healthineers, USA, which had no role in the study design, analysis, manuscript preparation or review, or decision to submit for publication. All other authors declare that they do not have a conflict of interest.

## Data Availability

Data will be made available on request.
