# Supplementary material for: Association between severe acute respiratory syndrome coronavirus 2 antibody status and reinfection: A case-control study nested in a Colorado-based prospective cohort study
Source: Prev Med Rep. 2023 Dec 1;37:102530. doi: 10.1016/j.pmedr.2023.102530 (PMC10776776; doi:10.1016/j.pmedr.2023.102530)
Supplement: Supplementary data 1 [file mmc1.docx]

**Online only Supplement to:**

**Association between severe acute respiratory syndrome coronavirus 2** **antibody status and reinfection: a case-control study nested in a Colorado-based prospective cohort study**

**Table of Contents:**

**Appendix Table A.1.** SARS-CoV-2 serologic antibody tests used in Colorado health system during study period: 2020-2022.

**Appendix Table A.2.** Baseline demographic and clinical characteristics based on electronic data available at start of or prior to study.

**Appendix Table A.3.** Self-reported behaviors and beliefs of enrolled survey and testing cohort participants (N=4,235).

**Appendix Table A.4.** Clinical outcomes among study cohort participants (N=4,235).

**Appendix Table A.5.** SARS CoV-2 antibody testing results among study cohort participants who had a primary SARS CoV-2 infection.

**Appendix Table A.6**. Results of sensitivity analyses of the association between SARS-CoV-2 antibody status and SARS-CoV-2 reinfection.

**Appendix Table A.1.** SARS-CoV-2 serologic antibody tests used in Colorado health system during study period: 2020-2022.

|  | Abbott Architect COV-2 | Siemens COV2-T Total ADVIA Centaur | Siemens COV2G Total ADVIA Centaur | Siemens sCOVG Total ADVIA Centaur |
| --- | --- | --- | --- | --- |
| Antibody type | IgG | IgM and IgG | IgG | IgG |
| Target | Nucleocapsid (N) | Receptor Binding Domain of Spike-1 protein (S) | Receptor Binding Domain of Spike-1 protein (S) | Receptor Binding Domain of Spike-1 protein (S) |
| Assay | CMIA | CLIA | CLIA | CLIA |
| Dates in use | April - July 2020 | July 2020 - February 2021 | February 2021 to end of study | February 2021 to end of study |
| Qualitative cut-off index | ≥ 1.4 | ≥ 1.0 | ≥ 1.0 | ≥ 1.0 |
| Semi-quantitative range | 0 to 10 and “>10” | 0.5-10 and “>10” | 0.5-20 and “>20” | 0.5-100 and “>100” |
| Affected by vaccination | No | Yes | Yes | Yes |

Abbreviations: CLIA, Chemiluminescent immunoassay; CMIA, Chemiluminescent microparticle assay

**Appendix Table A.2.** Baseline demographic and clinical characteristics based on electronic data available at start of or prior to study.

| Characteristics | Participants^a^  n=4235 | Non-respondents^a^  n=31993 |
| --- | --- | --- |
| Age, mean years (SD)^b^ | 49.4 (14.8) | 45.1 (17.1) |
| Female, n (%) | 2710 (64.0) | 17330 (54.2) |
| Ethnicity, n (%) |  |  |
| Hispanic | 780 (18.4) | 10107 (31.6) |
| Non-Hispanic | 3244 (76.6) | 18691 (58.4) |
| Unknown | 211 (5.0) | 3195 (10.0) |
| Race, n (%) |  |  |
| Black/African American | 120 (2.8) | 2254 (7.1) |
| Asian/Pacific Islander | 113 (2.7) | 1637 (5.1) |
| Native American | 21 (0.5) | 237 (0.7) |
| White | 3008 (71.0) | 14903 (46.6) |
| Multiple | 51 (1.2) | 346 (1.1) |
| Other^c^ | 235 (5.6) | 2246 (7.0) |
| Unknown | 687 (16.2) | 10370 (32.4) |
| Insurance, n (%)^b,d^ |  |  |
| Commercial | 2886 (68.2) | 21697 (67.8) |
| High deductible | 86 (2.0) | 498 (1.6) |
| Medicaid | 123 (2.9) | 2298 (7.2) |
| Medicare | 791 (18.7) | 4738 (14.8) |
| Private pay, self-funded, and others | 349 (8.2) | 2762 (8.6) |
| SARS CoV-2 RNA positive, n (%) | 1976 (46.7) | 11347 (35.5) |
| SARS CoV-2 antibody positive, n (%) | 373 (8.8) | 799 (2.5) |
| Resolved COVID-19 illness, n (%) ^e^ | 174 (4.1) | 730 (2.3) |
| Patient isolated/clinical suspicion, n (%) | 1147 (27.1) | 8975 (28.1) |
| No clinical or laboratory evidence/suspicion, n (%) | 565 (13.3) | 10142 (31.7) |

Numbers indicate column percentages

Abbreviation: SD=standard deviation

^a^ Recruited during the circulation of Alpha variant in Colorado.

^b^ Assessed on the survey invitation date.

^c^ “Other” category for race was based on electronic health record fields

^d^ If there was more than one insurance, the following hierarchy was employed: Medicaid, Medicare, commercial, high deductible, and others.

^e^ Resolved infection indicated patients who had clinical evidence of COVID-19 but may not have been tested due to limited test supplies early in the pandemic.

**Appendix Table A.3.** Self-reported behaviors and beliefs of enrolled survey and testing cohort participants (N=4,235).

| COVID-19 protective behaviors/beliefs | Mean item scores (SD)^a^ |
| --- | --- |
| I take steps every day to avoid getting COVID-19 | 4.4 (0.8) |
| Because of COVID-19, I… |  |
| …wear a mask every time I leave the house | 4.4 (1.0) |
| …believe everyone should wear a mask when in public | 4.4 (1.0) |
| …maintain 6 feet of distance from people when I go out | 4.4 (0.8) |
| …believe social distancing policies are necessary | 4.4 (0.9) |
| …avoid going to public places, gatherings, and crowds | 3.9 (1.2) |
| …avoid getting together with family and friends | 3.4 (1.2) |
| …believe it is too risky to go outside to walk or exercise | 1.6 (0.9) |
| …avoid using public or shared transportation | 3.7 (1.2) |
| …avoid going to places like health clinics and pharmacies | 2.6 (1.2) |

^a^ Response options were on a 5-point Likert scale corresponding to a scale of strongly disagree to strongly agree.

**Appendix Table A.4.** Clinical outcomes among study cohort participants (N=4,235).

| Characteristics | Participants |
| --- | --- |
| ***Primary Infection*** |  |
| Participants with ≥1 RNA test during follow-up (%) | 2650 /4235 (62.6) |
| Participants tested with ≥1 positive RNA test during follow-up (%) | 363 / 2650 (13.7) |
| Participants without RNA laboratory evidence of infection at baseline who had a positive RNA test during follow-up (%) | 214 / 2259 (9.5) |
| Participants without clinical or RNA or antibody laboratory evidence of infection who had a positive RNA test during follow-up (%) | 48 / 565 (8.5) |
| ***Reinfection*** |  |
| Participants with a primary infection and ≥90 days of follow-up with laboratory evidence of a reinfection (%)^a^ | 120 / 2033 (5.9) |
| Reinfection rate, per 100 person-months |  |
| Overall | 0.52 |
| Alpha | 0.04 |
| Delta | 0.11 |
| Omicron | 3.61 |
| ***Serology*** |  |
| Positive antibody test among participants with ≥1 antibody test during follow-up (%) | 2298 / 2894 (79.4) |
| ***Vaccination^b^*** |  |
| Not vaccinated | 610 (14.4) |
| Partially vaccinated | 103 (2.4) |
| Fully vaccinated but not boosted | 1394 (33.0) |
| Boosted | 2122 (50.2) |
| ***Deaths*** |  |
| All-cause (%) | 12 (0.3) |
| COVID-19 related (%) | 3/12 (25.0) |
| ***Cohort follow-up time*** |  |
| Mean follow-up days (SD) | 426.7 (127.4) |

^a^ Denominator includes those with at least 90 days of follow-up after primary infection; primary infection could have occurred prior to or during the study follow-up.

^b^ Denominator is 4229 study participants excluding the 6 participants where death date occurred early during the pandemic when the vaccines were not available or had just become available. All 6 who died had not received any vaccines.

**Appendix Table A.5.** SARS CoV-2 antibody testing results among study cohort participants who had a primary SARS CoV-2 infection.^a^

| Antibody Testing Outcomes | COV-2 nucleocapsid test | COV2T  spike test (IgG/IgM) | COV2G spike test (IgG) | sCOVG spike test (IgG) |
| --- | --- | --- | --- | --- |
| Antibody tests conducted after primary infection but before reinfection or vaccination, n (%)^a^ | 335 | 1919 | 873 | 575 |
| Time from RNA positivity to antibody test, mean days (SD) | 161.2 (85.7) | 104.1 (70.7) | 193.8 (88.4) | 143.7 (93.0) |
| Seropositive tests, n (%)^b^ | 229 (68.4) | 1802 (93.9) | 650 (74.5) | 495 (86.1) |
| Semi-quantitative index for seropositive tests, mean (SD), median | 3.7 (3.0), 2.8 | 7.8 (3.3), 10.0 | 5.4 (6.4), 2.5 | 12.6 (19.5), 4.6 |
| Index range^c^ | 0.01-10.0 | 0.5-10.0 | 0.5-20.0 | 0.5-100.0 |
| Participants tested ≥ 1 time who had ≥ 1 seropositive test (%) | 106 / 130 (81.5) | 877 / 941 (93.2) | 338 / 466 (72.5) | 355 /4 11 (86.4) |
| Participants with who had ≥ 1 seropositive test who seroreverted (%)^d^ | 29 / 70 (41.4) | 5 / 451 (1.1) | 27 / 163 (16.6) | 5 / 102 (4.9) |
| Mean days from first seropositive test to first seronegative test (SD) | 143.0 (47.3) | 90.4 (41.1) | 83.3 (49.3) | 48.2 (20.7) |

Abbreviations: COV-2, Abbott Architect SARS-CoV-2 nucleocapsid IgG test; COV2T, Siemens ADVIA Centaur SARS-CoV-2 Spike Total IgM/IgG test; COV2G, Siemens ADVIA Centaur SARS-CoV-2 Spike IgG test; sCOVG, Siemens ADVIA Centaur SARS-CoV-2 Spike IgG test, with wider range than COV2G; SD, standard deviation

^a^ Distributions reported are among individuals who had a positive RNA test but before receipt of a vaccine or reinfection except for the nucleocapsid test, which could have been after vaccination.

^b^ Participants could have been tested by more than one antibody test type and more than once.

^c^ Semi-quantitative.

^d^ Denominator is participants with ≥2 antibody tests after RNA positivity.

**Appendix Table A.6**. Results of sensitivity analyses of the association between SARS-CoV-2 antibody status and SARS-CoV-2 reinfection.

| Sensitivity Analyses | Analyzed Case Participants | Analyzed Controls | Adjusted matched odds ratio (95% CI)^a^ |
| --- | --- | --- | --- |
| (1) Excluding participants with the COV-2 nucleocapsid antibody test | 77 | 999 | 2.92 (1.44, 5.94) |
| (2) Excluding participants who were vaccinated after spike protein antibody tests | 64 | 761 | 4.23 (1.86, 9.62) |
| (3) Excluding participants in whom the antibody test was done ≥164 days before the index date | 40 | 441 | 3.96 (1.38, 11.37) |
| (4) Using an interaction term to assess the association by the timing of vaccination relative to the antibody test ^b^ | 79 | 1006 |  |
| Not vaccinated |  |  | 4.19 (1.28, 13.76) |
| Vaccination before the antibody test or on the same day |  |  | 6.30 (1.66, 23.94) |
| Vaccination after the antibody test |  |  | 1.08 (0.21, 5.48) |

Abbreviations: CI, confidence interval; COV-2, Abbott Architect nucleocapsid SARS-CoV-2 IgG test

^a^ All sensitivity analyses adjusted for significant variables in unadjusted analyses (ethnicity and household size) rather than all covariates due to smaller numbers of case patients.

^b^ This analysis excluded patients who had the COV-2 nucleocapsid antibody test and were vaccinated because the vaccine would not affect the test result.
